# Evaluation of novel immunocytokines that preferentially target high or intermediate affinity IL-2 receptors

**DOI:** 10.1186/2051-1426-2-S3-P118

**Published:** 2014-11-06

**Authors:** Zulmarie Perez Horta, Stephen Gillies, Margaret Boyden, Jaquelyn Hank, Alexander Rahkmilevich, Paul Sondel

**Affiliations:** 1 University of Wisconsin - Madison, Madison, WI, USA; 2 Provenance Biopharmaceuticals, Carlisle, MA, USA; 3 University of Missouri - Kansas City, Department of Pediatrics, Kansas City, MO, USA

## 

The parental hu14.18-IL-2 immunocytokine (ICp) is a fusion protein where one human IL-2 molecule is attached to each of the heavy chains of the intact humanized form of the anti-GD2 monoclonal antibody (mAb). Early clinical trials have shown that ICp treatment can lead to complete responses; however, dosing is limited by IL-2-induced toxicity. Evidence suggests that the anti-tumor efficacy of IL-2 is achieved through activation of immune cells expressing high-affinity IL-2 receptors (αβγ IL-2Rs) while IL-2 induced toxicity is related to over-stimulation of immune cells expressing intermediate affinity IL-2 receptors (βγ IL-2Rs). We have shown that ICp stimulates murine βγ IL-2R to a lesser degree than the human form, but is still able to induce potent anti-tumor effects in vivo. We believe this difference in the ICp selectivity profile for murine IL-2Rs may account for the successful retention of the anti-tumor effect and reduced IL-2 toxicity observed in mice. To study the effect of IL-2R selectivity on efficacy and toxicity we have created IC35 and ICSK, a novel generation of anti-GD2 ICs with varying levels of affinity for βγ IL-2Rs. These two new constructs, IC35 and ICSK, have the IL-2 molecules fused to the C-terminus of the light chains of the hu14.18 mAb rather than to the heavy chains. This modification limits access to a critical contact residue of IL-2 by the β-chain thereby hindering the ability of IC35 to bind to βγ IL-2Rs. In contrast to IC35, ICSK contains a mutated IL-2 protein with increased affinity for βγ IL-2Rs. Here, we evaluated the ability of IC35 and ICSK to bind and induce proliferation of mouse and human cells expressing αβγ or βγIL-2Rs. We found that IC35 and ICSK maintained binding and activation of both human and mouse αβγ IL-2Rs. In contrast, IC35 had a ~50-fold reduction or complete loss of its ability to stimulate proliferation of human and mouse cells expressing βγ IL-2Rs, respectively. Notably, ICSK had an increased ability to stimulate mouse cells expressing βγ IL-2Rs resulting in similar receptor activity and selectivity that IC35 has for human immune cells. Overall, our data indicate that ICSK can be used in mouse models to study the effects of over stimulation of βγ IL-2Rs and its relation to IL-2 induced toxicity. In addition, IC35's reduced ability to stimulate human βγ IL-2Rs suggests it may be a candidate for retaining anti-tumor activity with less dose-limiting IL-2 toxicity in clinical trials.

